# The COVID-19 pandemic as a fortuitous disruptor in physical education: the case of active homework

**DOI:** 10.3934/publichealth.2022029

**Published:** 2022-04-29

**Authors:** Richard Bailey, Claude Scheuer

**Affiliations:** 1 Centre for Academic Partnerships and Engagement, University of Nottingham Malaysia, Semenyih, Selangor, Malaysia; 2 Institute for Teaching and Learning, Department of Education and Social Work, University of Luxembourg, Esch-sur-Alzette, Luxembourg

**Keywords:** schools, homework, health-enhancing physical activity, COVID-19, lockdown, online

## Abstract

Measures devised to contain the COVID-19, including isolation, social distancing, and quarantine, have profoundly affected people's lives around the world. One of the consequences of these actions has been a general reduction in the habitual daily physical activity among children and young people for whom schools represent the major setting for the promotion of sports, physically active play, movement skills learning, and other activity supportive of healthy, active lifestyles. Whilst acknowledging the seriousness of these changes, and their concomitant health risks, we suggest that COVID-19 offers an opportunity to think again about important features of school-based activity promotion in light of new lessons learnt during lockdown, emerging technologies, and adapted pedagogies. In these specific cases, COVID-19 could be judged a “fortuitous disruptor” to the extent that it has opened a window of opportunity to schools and teachers to reflect on their assumptions about the scope, content, and delivery of their curricula, and on the new professional knowledge that has emerged. Active Homework, or physical activity-related tasks assigned to students by teachers that are meant to be carried out before, after and away from school, that students can do on their own or with family members, is not a new idea, but the enforced changes to school provision have made it considerably more common since the pandemic. Perhaps Active Homework is a concept worth retaining as schools start to return to “normal”? We offer a typology of Active Homework, and examine opportunities to expand, extend, and enhance physical education and physical activity opportunities by breaking down the presumed boundary between school and home. In conclusion, we suggest that Active Homework is worth exploring as a potentially valuable approach to enhancing the quantity and quality of students' school-based health-related physical activity. If so, considerably more research and curriculum development is needed.

## 1. Introduction

“The dogmas of the quiet past are inadequate to the stormy present. The occasion is piled high with difficulty, and we must rise with the occasion. As our case is new, so we must think anew and act anew.” [1]

COVID-19 is undoubtedly one of the most disruptive events confronting communities and states in recent times. The measures implemented to curb the pandemic have modified daily life and have introduced marked changes in people's behaviour. Understandably, the dominant narrative from this situation has focused on the harm it has done to individual and community well-being, including considerable economic costs [2]. Health costs, as will be discussed later in this article, are less clear-cut, although there is no doubt that billions of people's lives have been negatively affected, both directly due to the virus and indirectly due to its concomitant effects in terms of negatively impacted self-reported diet, HEPA, sleep, mental health, and access to health services [3]–[5]. Borrowing an increasingly popular business term, many commentators have described the pandemic as a “disruptor”. A disruptor, in these contexts, refers to an event that challenges assumptions about current principles and practices associated with a situation, forcing a reappraisal of fundamental aspects of that situation. Technology, politics, science, medicine, working practices, disasters, and many other factors can become disruptors insofar as they can radically alter key aspects of our people's lives and the ways in education systems seek to respond [6]. There is no doubt that the COVID-19 pandemic and the subsequent restrictions of social contact has disrupted education [7], healthcare [8], and other domains.

This article argues that whilst we should not overlook the real human costs of COVID-19, we should remain open to its potentialities. As President Lincoln advised, when the dogmas of the past are found to be inadequate, we must think anew and act anew [1]. COVID-19 and its consequences have exposed our educational dogmas, and schools and teachers have shown alternative ways forward.

## 2. COVID-19 and children's schooling

Children and young people are among the least vulnerable to the effects of the COVID-19 virus of the total population [8],[9], yet they have been among the groups most negatively affected. According to OECD [10], more than 1.5 billion students from 188 countries were locked out of their schools, and while many of these students and their families were able to find alternative routes to learning, others were not. This crisis has in many ways exacerbated existing inequalities in education. Socially marginalised groups, such as minority ethnic groups and low socio-economic status families, felt the impact of school shut-down most strongly, often because they lacked access to digital learning technologies or the social support networks necessary to compensate for the suddenly impoverished pedagogical environment. The current generation of school students risks losing more than $17 trillion in lifetime earnings, or about 14 per cent of today's global GDP, as a result of COVID-19 pandemic-related school closures [11].

**Figure 1.**
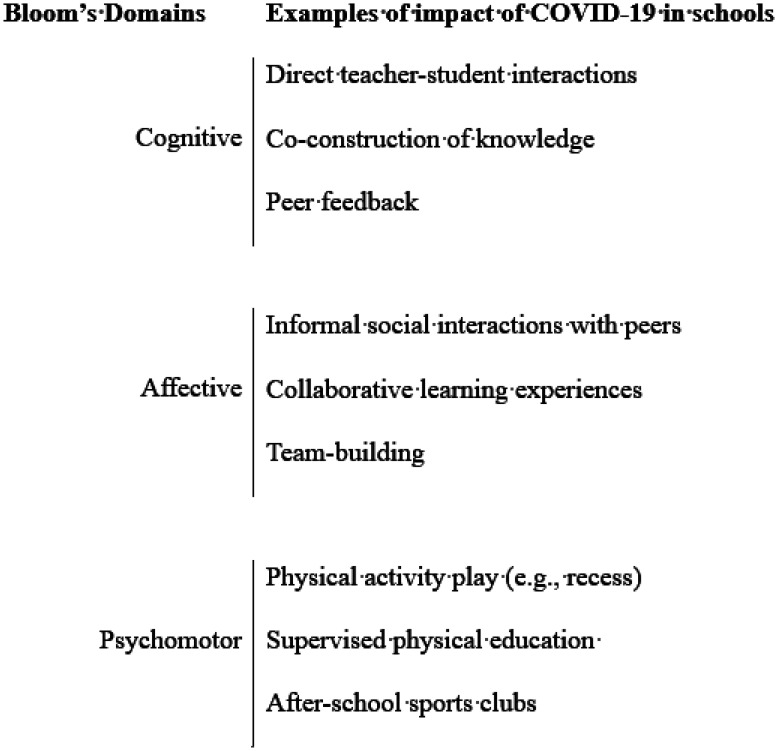
COVID-19's impact of educational domains.

COVID-19 has disrupted very many aspects of education. Indeed, it has forced all involved—teachers, students, parents, leaders—to reconsider some fundamental assumptions about schools and schooling. Education, as Bloom [12] famously articulated, is inherently multi-dimensional and aspires to learning and development in several domains, and each of these was affected by the pandemic and the subsequent responses by governmental and educational agencies. Figure 1 offers an indication of the extent of COVID-19's impact on education.

There is little doubt that “Cognitive” outcomes dominate in most education systems around the world, and in some countries (e.g., many Asian states), there is a relatively narrow focus on academic knowledge. However, there has been a clear attempt by international agencies, such as UNESCO and OECD [13] to broaden the scope of national system to give parity to non-cognitive outcomes, such as social development, emotional awareness, and health. These calls are often framed within discussions of education for well-being [14], and health often figures prominently within such accounts. This is hardly surprising as well-being-based account of education are usually justified in terms of the importance of students' quality of life, both during schooling and in later life. To be clear, the movement to well-being-based education does not carry with it any claim that non-cognitive outcomes are more important than cognitive outcomes. Rather, the implication is that more inclusive educational frameworks that include a range of goals are better-suited to the demands of contemporary societies than narrow ones.

The traditional notion of a school as a physical space, populated by students and teachers, following relatively rigid curricula was abandoned with little notice in most countries. According to the OECD [10], the interference of COVID-19 in terms of school closures increased with the students' level of education, representing 56% of total teaching days at the high school level. Eventually, online learning emerged as a “new normal” in almost all countries, and students and teachers had to learn to adapt to a new type of schooling in which “homework” and “work” became somewhat synonymous.

## 3. COVID-19, health, and HEPA

There are health and well-being costs associated with the effects of the pandemic on children and young people. Patterns of HEPA, for example, were radically changed following the COVID-19 outbreak and concomitant shutdown of organised sports and public sports facilities, and the imposition of physical distancing measures and contact restrictions in most countries, allowing, for example, no more than two people from different households to meet in public space, led to significant changes in daily routines and their opportunities for being active. This resulted in a complete shutdown of organised HEPA and sport. Reductions in daily energy expenditure, uncompensated energy intake, altered sleep, and a decrease in levels of voluntary HEPA are likely to exacerbate the on-going public health concerns about low levels of HEPA and the high prevalence of sedentariness. In addition, social isolation affects psychological health. The COVID-19 lockdown also affected the emotional health and habits of children and adolescents. Boredom, irritability, and reluctance were more present during the lockdown.

There seems little doubt that school closures and the concomitant restrictions (social distancing, quarantining, etc.), negatively impacted the lifestyle activities of children and adolescents across the day [15]–[17], although these changes were mediated by contextual factors, such as the physical environment [18],[19]. Evidence suggests that social restrictions led to the increase in sedentary behaviour [20],[21] and decreased opportunities for children and adolescents to engage in HEPA [22]. For example, a study carried out in China [23] showed that compliance with World Health Organisation HEPA guidelines—“at least an average of 60 minutes per day of moderate-to-vigorous-intensity, mostly aerobic, HEPA, across the week” (p. 1)—fell from 60% before to 17.7% during the pandemic. Studies from other countries have reported similar patterns of decreases in HEPA and increased in sedentary behaviour during periods of lockdown, compared to before and after lockdown (the Netherlands [24]; Austria [25]; Japan [26]; Canada [27]). These behaviour changes are known to be detrimental to long-term cardiometabolic and psychological health outcomes in the general population [28], and there is convincing evidence that they can also develop into long-term poor health outcomes in children and adolescents [29],[30]. For example, findings from studies of earlier disasters have reported significant decreases in HEPA in children and adolescents over three years following a disaster [31].

Of course, the closure of schools is only partially responsible for these changes. Children and adolescents' engagement in HEPA extends beyond the school grounds, and for many children, schools represent a relatively small proportion of daily HEPA [32]. Nevertheless, in many countries, schools remain hubs of HEPA and sporting activity. Consistent with the growing evidence in favour of “settings” approaches to health promotion, in which foci are shifted from individual-based risk orientation and disease to social factors that support human health and well-being [33], schools have been identified as an important context for the development of physical skills and the provision of HEPA in all children and young people [34]. It could be argued that movement towards the inclusion of public health within the intended goals of education is a challenge to traditional approaches. However, narrow, knowledge-focused curriculum frameworks are actually relatively recent developments, and, historically, a public health element of schooling has been the norm, not the exception [35].

PE plays an important role in school-based HEPA promotion as the only protected, regular, supervised setting for HEPA during the school day. Students are more active during PE lessons than in any other context, although they generally fail to achieve 50% of lessons at moderate-to-vigorous-physical-activity [36]. Closure of schools significantly affected the provision of PE, although this was mediated by school systems and the initiative of individual teachers and schools [37]. Those teachers who tried to continue PE lessons using distance learning technologies struggled to adapt their pedagogical practice to non-contact environments [38]. Nevertheless, the broadly inclusive character of public schooling means that it is well-placed to address the enormous disparities in access to these opportunities based on family income, technology access, home space, and neighbourhood safety and traffic volume [39],[40].

## 4. COVID-19 as a fortuitous disruption

While the impact of the COVID-19 pandemic and its related social restrictions are yet to be fully understood, it seems unarguable that it has generally led to a reduction in children and young people's HEPA and an increase in their sedentary behaviour, and this pattern is cause for concern as inactivity during this period is associated with a wide range of harmful consequences [41]. Yet, the situation is not all bad. Studies from several countries have revealed an interesting and potentially important phenomenon: overall decreases in some forms of HEPA but increases in others. For example, a German study reported reduced sports participation but increased habitual physical activities during school closure, including greater uptake of gardening, housework, cycling or walking [42]. Other studies report an increase in non-organised activities, such as workouts at home, or jogging, and other forms of habitual HEPA, such as going for a walk or playing outside remained allowed if done under certain rules [43]–[45].

Children and young people's HEPA are not the sole domain of schools, and evidence suggests that many engage in substantial amounts of health-related HEPA, both with their families and in their free time [32]. However, it is also known that a growing number of young people, especially those from lower socio-economic backgrounds and other marginalised groups [46], rely on schools as settings for HEPA, especially in terms of basic skills learning, adult guidance, and structure. So, taking the school-aged population as a whole, schools are the main societal settings for being physically active, developing new physical skills, and receiving health-promoting messages, whether through PE lessons, other in-school opportunities, or after-school activities [46],[47]. It is interesting to note, then, that many teachers sought to address the absence of school-based provision through the introduction and extension of digital technologies in support of school-related HEPA during the lockdown, including video conferences or tutorials [38],[48], with some evidence of increases in behaviours promoting students' self-regulated HEPA using these tools [37].

We suggest that these, albeit tentative, findings point towards some positive among the negativities of the COVID-19 pandemic. Lockdown forced schools to rethink their support of their students, often resulting in remarkable problem-solving and innovations. So, the pandemic can be seen as a catalyst for change, or a “fortuitous disruptor”, as it offered (or forced) schools and teachers to rethink their assumptions about the nature of schooling. Before COVID-19, the default conception of a school was equated with buildings, classrooms, and other physical capital in which teachers taught students in enclosed and time-regulated lessons. The attempts to maintain schooling throughout the COVID-19 pandemic, primarily through digital technologies, have challenged this equation. Aside from the other potential developments that occurred during the lockdown, it seems clear that online and offline education cannot be thought of as discrete educational experiences. Rather, they can be better framed as parts of a holistic system [49].

One way of thinking about fortuitous disruptors is with the help of the German philosopher, Hans-Georg Gadamer [50], and his claim that all experience can be subdivided into two types: Erlebnis, which could be called “ordinary experience” or experience that conforms to our expectation and confirms it; and Erfahrung, which is “genuine experience” or experience that occurs as new and disrupting. The power of Erlebnis lies in the fact that the world generally makes sense to us because it conforms with our expectations and prejudices. In contrast, Erfahrung is always negative in the sense that “if a new experience of a thing occurs to us, it means that we have previously not seen that thing genuinely and now we know it better. Thus, the negativity of Erfahrung has a curiously productive meaning” [50] (p. 347). The relevance to the present discussion is that Gadamer highlights the potential transformative force of challenging experiences, such as the lockdown, by presenting a “new normal” and underpinning the cosy certainties of the past.

Of course, radical change, such as has been witnessed during the pandemic, is not inherently generative. Years before COVID-19, Gard and Pluim [51] cautioned about the potentially harmful effects of a shift to digital PE. Some of their concerns regarding the use of digital technology to measure, record, disseminate and analyse the results of school-based student fitness testing seem entirely reasonable, and the threat that such programs could become PE's version of ‘high-stake testing’ is all the more worrying in light of the absence of critical examination of its implications for the definition of the subject. Other reasonable concerns related to the imposition of ‘touchless’ classes, where bodies exist mainly through screens [32], representing a change too far in how PE is presented to students. However, these calls should be understood as concerns about, on the one hand, reductionist redefinitions of PE, and necessary compromises in a unique situation, on the other hand, rather than falsifications of alternative pedagogies, per se. Nobody, we suspect, is advocating that traditional, face-to-face PE be abandoned in favour of digital curricula. What is being suggested is that traditional approaches become supplemented by innovative and relevant pedagogies that break down the online/offline and in-school/out-of-school divisions. Discussions on such changes are already familiar to many researchers and practitioners of PE. We will briefly consider two of these approaches: “Active Schools”, and the “Theory of Expanded, Extended, and Enhanced opportunities for youth HEPA promotion”.

The concept of the Active School is a radical departure from traditional approaches to HEPA promotion that seeks to reconcile the potential of the school as a unique setting for the promotion of healthy behaviours, with the barriers presented by conventional approaches [52]. The active (or moving) school seems to have been first proposed by the Swiss educational theorist Urs Illi [53] in the early 1980s. Illi advocated bringing more movement into the traditional “seated school”. The Moving School concept has been quite influential in German-speaking countries; initially applied to the elementary phase of schooling, the moving school idea has recently been extended into high schools, as well [54]. A similar concept was proposed in an English-speaking setting by Fox [55] in the United Kingdom, who called for a more ecological model of school-based PA promotion that went beyond the curriculum. Fox and Harris [56] argued that the traditional focus on physical education classes provided only one part of the solution since it represents less than two per cent of children's waking time. From a whole-school perspective, many elements can either promote or inhibit the adoption of an active lifestyle, and development initiated in the taught curriculum can either be supported or undermined by external influences (e.g., peers and family socialisation, or contradictory school policies). This was the stance taken by the US Institute of Medicine: “Clearly schools are being underutilized in how they provide opportunities for HEPA for children and adolescents. A whole-of-school approach that makes the school a resource to enable each child to attain the recommended 60 minutes or more per day of vigorous or moderate-intensity HEPA can change this situation” [61] (p. S–6). To this, we would add one qualification, namely that the “school” need not be understood as merely a physical building, but rather a conceptual space for learning that reaches out into the local community and home. Under such an approach, all of a school's components and resources operate in a coordinated and dynamic manner to provide access, encouragement, and programmes that enable all students to engage in sustained periods of relatively vigorous HEPA every day by utilising all segments of the school day, including travel to and from school, school-sponsored before- and after-school activities, recess and lunchtime breaks, PE lessons, and classroom instructional time. Importantly, they are framed within contextual or socio-ecological perspectives that acknowledge the need for the engagement of all school stakeholders (such as students, teachers, parents and the wider community) [62].

The “theory of expanded, extended, and enhanced opportunities for youth HEPA” (TEO) [63] is a pragmatically orientated theory to the extent that it generalises and formalises practices that are already happening in schools but making sense of them within a theoretically robust umbrella concept. These approaches typically involve: “(a) the expansion of opportunities for youth to be active by the inclusion of a new occasion to be active, (b) the extension of an existing HEPA opportunity by increasing the amount of time allocated for that opportunity, and/or (c) the enhancement of existing HEPA opportunities through strategies designed to increase HEPA above routine practice” (p. 1). This is consistent with existing scientific theories of health promotion, such as the socio-ecological framework [62] but takes as its starting point the realities of schools. TEO is consistent with, and somewhat augments, the Active Schools concept by highlighting specific mechanisms through which teachers could supplement HEPA opportunities both within and outside of schools.

These two approaches are different in intent, as the former offers an institution-level framework for policy development and the latter presents what could be called a “didactic” theory of provision [64], but they share a set of claims. First, if schools are going to properly contribute to the support of students' HEPA, they must abandon traditional reliance on PE lessons only in favour of a more holistic and multi-contextual approach. Second, this expansive perspective requires teachers and schools to explore new settings for promoting and guiding HEPA by identifying real-world opportunities. Third, such innovations are unlikely to happen without challenging norms of practice, and, as Gadamer argued, seeing what has previously not been seen. The disruption caused by the COVID-19 pandemic proves to be fortuitous if it helps establish a “new normal” in the short-terms that reveals a possibility for the long-term.

## 5. Active homework

**Figure 2.**
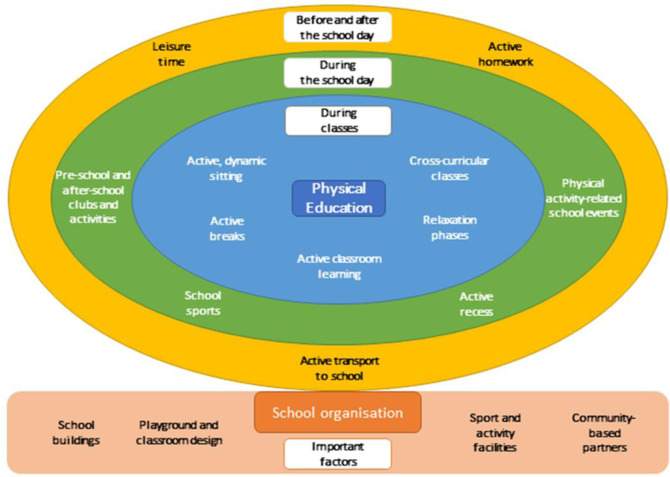
The active school concept [49] (p. 174).

Inherent within the Active Schools concept is the idea that all of a school's components and resources operate in a coordinated and dynamic manner to provide access, encouragement, and programmes that enable all students to engage in HEPA each day. Advocates have proposed several proposals for specific elements of this model. Figure 2 suggests one way of conceptualizing these elements.

A recent review [36] examined many of the elements proposed by the Active School concept focusing particularly on their contribution to the World Health Organisation's guidelines on HEPA and sedentary behaviour [65]. The review reported that “school-based interventions have been found to have significant effects on young people's physical activity and sedentary behaviours, although the effects have varied considerably. The key variable in determining the positivity and scale of the outcomes of participation in physical activities is the social environment in which they take place” [36] (p. 8). It also found some settings that have been relatively under-researched and under-utilised, and perhaps the clearest example of this was “Active Homework” (AH). Calls for further research and stronger theoretical discussions have been made from writers in Europe [57] and North America [58]–[60].

The Active Schools concept and the theory of expanded, extended, and enhanced opportunities share a motivation to address the perceived need to improve children and young people's HEPA levels, capitalising on the distinctive characteristics of the school setting [33],[66]. AH has been proposed as a possible way of contributing to this goal by extending the time available for schools to influence the health behaviours of students [67]. There is an absence of working definitions of AH in the literature, however, some insight can be found in the general educational literature. For example, Cooper [68] defined homework as “tasks assigned to students by school teachers that are meant to be carried out during non-school hours” (p. 7), or teacher-assigned tasks to engage students in independent and effective studying [69]. Implicit in these definitions is a recognition that students must manage homework assignments by engaging in various self-regulation processes such as planning, managing time, finding a suitable place to work, and motivating themselves. PE differs from most school subject areas, both in terms of its movement-based content, and the sometimes porous boundary between educational and recreational activities. With the possible exception of the arts, PE is unique among curricular domains in that much of its content, from basic movement skills and physically active games during the early stages of schooling to sports, swimming, and outdoor activities for older students, are voluntarily engaged in free time, whether individually, with peers, or with family members. In addition, many of the basic contextual requirements of HEPA (open spaces, sports clubs, gardens, etc.) are freely available for most children. Perhaps this goes some way to explain the phenomenon reported above where some students are engaged in greater engagement in certain types of physical activities during school lockdowns?

We propose the following working definition:

*Active Homework is physical activity-related tasks assigned to students by teachers that are meant to be carried out before, after and away from school, that students can do on their own or with family members*.

So, AH consists of tasks that are linked in one way or another to movement activities, at home. Activities can be designed for students to apply and practice the skills learnt in PE lessons and might take place at home (with or without parents' or sibling involvement), and in nearby sporting environments or facilities [70]. Extending HEPA in this way increases time and opportunities for movement, and it has an additional advantage for less able students who can practice skills in a less exposed setting [71].

There is a wide range of potential opportunities to constitute AH, including learning new and challenging movement tasks such as learning juggling with balls or cloths, introducing relaxation techniques, planning active breaks during other homework tasks (similar to active breaks during classroom lessons), or simply doing homework in a physically active way (reading when walking, balancing, standing, etc.). Table 1 outlines possible AH content.

This definition does not include in-school teaching or coaching, extra-curricular, school-based activities (e.g., clubs, sports), or home study courses offered by other providers. It also excludes participation in sports groups or clubs that are unconnected to schools, although these might become involved as settings for specific homework activities (e.g., observation or reflection tasks).

Evidence of the implementation of AH, in the sense suggested above, is limited [33]. The available literature suggests that the most common specified goal of AH has been to create additional opportunities for physical activities [72]. Another posited rationale of AH is to encourage students to become more familiar with their local environments and available facilities as a foundation of helping make physical activities part of their lifestyles [73]. This is in contrast to the traditional purpose of homework, which has been to enhance a student's level of academic achievement [60]. However, several educational systems now include theoretical or conceptual content within PE curricula, such as the promotion of knowledge about health-related HEPA and fitness, healthy lifestyles (in Australia, New Zealand, England, Wales, Scotland, Northern Ireland, Ireland, and others) [74].

**Table 1.** Categories and examples of AH.

Relation to school subjects	Categories of AH	Examples of AH
AH related to PE classes	Skill learning and practice	Learning new skills; practising and/or adapting skills already learnt in PE classes; learning new skills in the context of other clubs (e.g., martial arts skills).
	HEPA – being more active	Active travel around the environment; increasing steps using a pedometer; hill walking with the family.
	PE knowledge – studying theoretical and conceptual knowledge	A basic exercise physiology and anatomy; history of sport; planning, evaluating and reflecting tasks or performance.
AH related to other classes or school-level activities	Practice of physically active breaks during other homework tasks	During other homework tasks, students have an active break after each work sequence of 20'. They practice one of the active breaks they practice regularly during classroom lessons.
	Practice of relaxation techniques during other homework tasks	During other homework tasks, students have a relaxation break, implementation a technique they practice regularly during classroom lessons once they start to feel unconcentrated (e.g., stretching, yoga, ...).
	Practice of physically active learning during other homework tasks	During other homework tasks like e.g., reading, students are not sitting at a desk or a table, but are standing, walking or balancing in their room.

The greatest amount of research attention in this area has been given to the examination of students' attitudes [75], and the findings are ambiguous. Research in Finland [67] found that students can find homework connected to PE lessons enjoyable and beneficial. For example, students reported positive responses to homework that included practising with family members. However, a study based in the US [76] reported a mixed response from students to a “Cultural Studies unit” linked to the PE programme. Some students endorsed opportunities for taking responsibility for their learning, discussions of social issues, such as gender, body image. and sports media, and content that drew a connection between life and out of school. Others resisted the programme, and there was a generally low compliance rate among the group as a whole, with interviewees claiming that PE is not a “real class” (p. 255), and that homework was unreasonable for such that class. The authors of the study [76] attributed this resistance to lack of clarity of the purpose of the “Cultural Studies unit”, and unsettled feelings for the new expectations. Other studies based in the US [77],[78] and Israel [79] reported similar resistance to AH from students. It is not possible to explain these starkly different reactions to AH as these studies were generally with small samples and in single school settings. Discussions of the importance of perceptions of relevance seems to warrant further investigation, and, of course, the different existing PE curricula might have shaped students' expectations of the subject area, its content, and delivery.

An insight into effective planning of AH comes from the “structured days hypothesis” [80] which suggests health-related behaviours (HEPA, sedentariness, diet) are more beneficially regulated during relatively structured days. Conventional schools, of course, are the paradigmatic examples of structured days, occupying and organising a large part of children's days. Extrapolating findings from summer camps and other organised out-of-school events, which seem to be effective in either slowing or reversing the gradual drift into sedentariness and weight gain [81], researchers have argued that health-related behaviours (HEPA, sedentariness, diet) are more beneficially regulated during relatively structured days (e.g., schools days; residential camps) than during less structured days (e.g., long holidays; weekends). In addition, children typically engage in lower levels of HEPA and more sedentary time on weekend days compared to school days [82]. Further support for this hypothesis indicates that children and young people, especially the less active [82], are more active when their days are timetabled to some extent. In some ways, the COVID-19 pandemic presented an unfortunate opportunity to test the structured days hypothesis, as children and their families suddenly found themselves robbed of the normal frameworks of school and work. Generally, declining patterns of HEPA, as already discussed, are consistent with the theory. Considering this theory in the light of the empirical findings from AH initiatives suggests that teachers and schools would be advised to strike a balance between the self-regulated opportunities offered by homework and the structure needed to help students maintain motivation and engagement.

Many commentators have noted the potential value of incorporating digital learning as a way of enriching student learning whilst monitoring their compliance with expectations (e.g., [83],[84]). This idea received unprecedented testing and, to some extent, endorsement during the pandemic [85], and there has been a significantly accelerated growth in the development of online resources since the first stages of lockdown [46]. PE professionals have been active in developing alternative pedagogies for maintaining student engagement with their curricula [35],[47], and initial findings are encouraging in terms of supporting healthy behaviours and self-regulated HEPA [36]. If these changes are sustainable, though, both teachers and students will need to be prepared. As has become apparent, there is no guarantee that students will necessarily welcome a radical change to their PE experience without proper initiation. Many teachers will certainly require professional development to capitalise on the potential opportunities offered by new technologies, especially if they are to be employed in previously under-utilised settings, such as at home.

## 6. Conclusions

This There is an old Irish joke about two walkers asking a farmer how to get to Dublin. “Dublin?”, the farmer replied. “Well, I wouldn't start from here!”

The different manifestations of PE around the world, like every other aspect of educational policy, are the results of numerous, often mutually exclusive, pressures [86]. Unique social, cultural, economic, and political contexts affect specific policy or reform implementation in different localities while globalising forces push towards conformity [87]. So, it seems somewhat counter-intuitive that PE has historically tended to be slow to respond to external forces and changing contexts [88], often resorting to variations of existing models, rather than radical change. The COVID-19 crisis did not present a safe option, and professionals were forced to explore new ideas and practices to engage and motivate their students to keep moving. This obvious disrupted PE as it did other subject areas, and schools as a whole, but the enforced disruption also presented a unique opportunity to re-assess the nature of PE and the HEPA experiences we want for our children in the future [89].

Whatever other goals and content might be considered for inclusion within PE, it seems unarguable that health-related PA must form a central feature. Current schooling structures, however, generally make the sustainable impacts of PE on PA negligible due to simple lack of time. In this regard, we suggest that AH is worth exploring as a potentially valuable approach to enhancing the quantity and quality of students' school-based health-related PA. So, COVID-19 might turn out to be precisely the fortuitous disruptor the PE needs as it moved forward.
